# Sinus Node Dysfunction due to Occlusion of the Sinus Node Artery during Percutaneous Coronary Intervention

**DOI:** 10.1155/2021/8810484

**Published:** 2021-03-30

**Authors:** Ofir Koren, Dante Antonelli, Ranya Khamaise, Scott Ehrenberg, Ehud Rozner, Yoav Turgeman

**Affiliations:** ^1^Heart Institute, Emek Medical Center, Afula, Israel; ^2^Bruce Rappaport Faculty of Medicine, Technion Israel Institute of Technology, Haifa, Israel; ^3^Internal Medicine B, Emek Medical Center, Afula, Israel

## Abstract

**Background:**

Sinus node artery occlusion (SNO) is a rare complication of percutaneous coronary intervention (PCI). We analyze both the short- and long-term consequences of SNO.

**Methods:**

We retrospectively reviewed 1379 consecutive PCI's involving RCA and Cx arteries performed in our heart institute from 2016 to 2019. Median follow-up was 44 ± 5 months.

**Results:**

Among the 4844 PCIs performed during the study period, 284 involved the RCA and the circumflex's proximal segment. Periprocedural SNO was estimated by angiography observed in 15 patients (5.3%), all originated from RCA. The majority of SNO occurred during urgent and primary PCIs following acute coronary syndrome (ACS). Sinus node dysfunction (SND) appeared in 12 (80%) of patients. Four (26.6%) patients had sinus bradycardia, which resolved spontaneously, and 8 (53.3%) patients had sinus arrest with an escaped nodal rhythm, which mostly responded to medical treatment during the first 24 hours. There was no association between PCI technique and outcome. Three patients (20%) required urgent temporary ventricular pacing. One patient had permanent pacemaker implantation. Pacemaker interrogation during follow-up revealed a recovery of the sinus node function after one month.

**Conclusion:**

SNO is rare and seen mostly during angioplasty to the proximal segment of the RCA during ACS. The risk of developing sinus node dysfunction following SNO is high. SND usually appears during the first 24 h of PCI. The majority of SND patients responded to medical treatment, and only in rare cases were permanent pacemakers required.

## 1. Introduction

Sinus node supply by the sinus node artery (SNA) originates from RCA in almost 60% of cases and the circumflex artery in 30–40% [1]. In about 10–11% of cases, the sinus node has a dual blood supply by RCA and circumflex arteries [1].

Side branch occlusion of coronary arteries is a well-known complication of PCI (percutaneous coronary intervention), especially after stent deployment [2–6]. Previous reports describe iatrogenic occlusion of the sinus node artery in cases of proximal RCA (right coronary artery) and circumflex artery (Cx) intervention [7–11].

Several case reports and small clinical trials described the short-term consequences of sinus node artery occlusion [12–15]. Therapeutic approaches and long-term outcomes are still unclear.

### 1.1. Study Rationale

The study aims are to analyze SNO incidence during PCI on the RCA and circumflex artery proximal segments, patients' clinical characteristics, and immediate and late outcome of SND as reported.

## 2. Methods

We conducted an observational cohort study in Emek Medical Center, a general, 500-bed teaching hospital in Israel's northeast region, belonging to the Clalit Health Services. We collected data from patients who underwent PCI from June 2016 to June 2019 who met the study inclusion criteria (Table 1).

Patients with evidence of sinus node dysfunction before the PCI, such as inappropriate sinus bradycardia, bradycardia-tachycardia syndrome, sinus pause or arrest, and sinoatrial (SA) exit block, were not included in the study. Patients with supraventricular arrhythmias (atrial tachycardia, atrial flutter, or atrial fibrillation), a cardiac implantable electronic device (CIED), and patients who needed coronary artery bypass graft surgery were also not included in the study.

**Figure 1:**
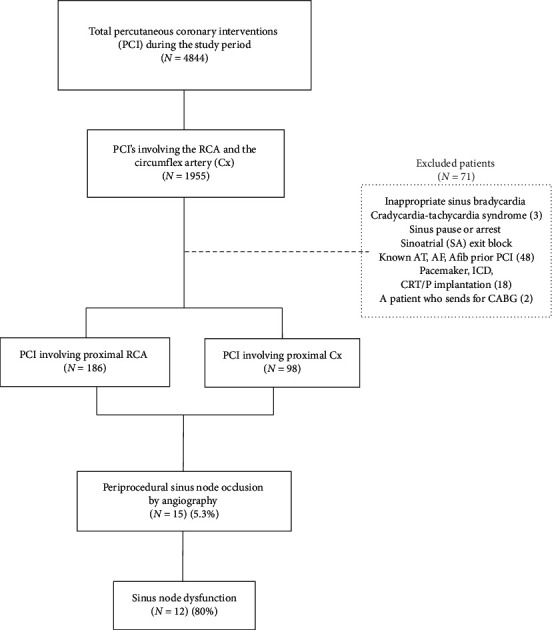
Study plan. AT, atrial tachycardia; AF, atrial flutter; AFib, atrial fibrillation; PCI, percutaneous coronary intervention.

We used computer-based data mining to identify coronary angiography involving the right coronary artery and circumflex artery proximal segments. Three senior cardiologists analyzed consecutive PCIs to determine SNO by angiography. Independent authors reviewed all personal medical records, ECG strips, and catheterization reports for documented sinus node dysfunction (Figure 1).

Emek Medical Center is a regional hospital that belongs to Clalit Health Services. We used a computerized scan of the hospital and Clalit Health Services databases for long-term follow-up. When there was not sufficient information regarding outcomes, an experienced physician reviewed the medical files and, if required, contacted the patient.

### 2.1. Ethics

The Emek Medical Center Ethics Committee approved the study following the Helsinki Convention. The IRB waived the informed consent due to patient data confidentiality and the study's methodology (No. EMC-113-19).

### 2.2. Sample Size

The sample size was calculated based on a previously reported SNO incidence of approximately 25% of PCI involving RCA and Cx's proximal segment. To achieve a confidence level of 95%, a margin of error of 5%, and a response distribution of 50%, we required a minimum sampling size of 102 PCIs. Preliminary analysis revealed a lower than expected SNO incidence, which required us to increase the sample size.

### 2.3. Statistics

We described categorical variables using frequencies and percentages and continuous variables using mean ± standard deviation. We used the *T*-test (or alternative Wilcoxon two-sample test) for continuous variables and multivariable models and one-way ANOVA to estimate SND appearance. A *P* value < 0.05 and CI 95% were considered significant. SAS 9.4 software was used for statistical analysis.

## 3. Results

We performed 4844 PCIs during the study period. 15% (284) of all RCA and circumflex artery interventions involved the proximal segment (186 and 98 for RCA and Cx arteries, respectively). The sinus node artery was originated from the proximal segment of the RCA in 181 (67%) cases and the proximal segment of the circumflex artery in 68 (24%) PCIs.

Sinus node artery occlusion was observed in 15 (5.3%) patients and only during angioplasty to the RCA's proximal segment. None of the cases of SNO involved the circumflex artery. Patients' average age was 69.58 ± 8.85 years, and 86% of them were male. More than half of them were smokers who had hypertension, hyperlipidemia, and diabetes mellitus (0.6, 0.73, 1.0, and 0.53, respectively). SNO was observed during primary and urgent PCI in almost 90% of cases (0.6 and 0.27 for immediate and urgent PCI, respectively) and mostly during inferior ST-elevation myocardial infarction and acute coronary syndrome (Table 2).

**Figure 2:**
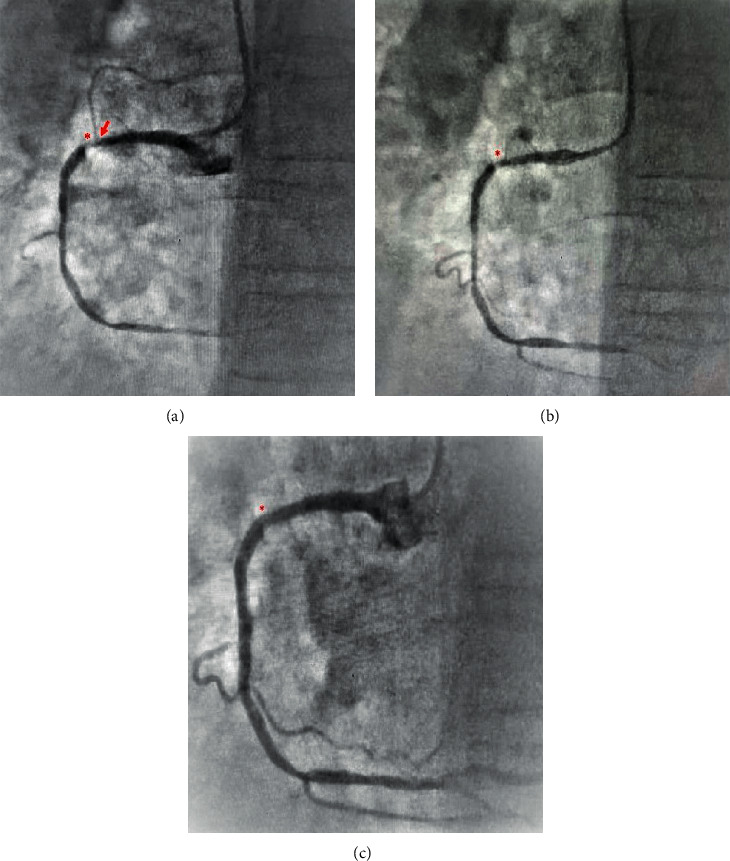
(a)–(c) Sinus node artery before and after PCI. (a) Right coronary angiography showing 95% narrowing in the proximal segment (red asterisk). Note the sinus node artery (SNA) originated from the proximal segment (red arrow). (b) RCA following ballooning. (c) Proximal segment stenting. Note the angiographic disappearance of the SNA (red asterisk).

Sinus node dysfunction (SND) following SNO appears in 12 (80%) of patients. Fours patients (26.6%) had transient sinus bradycardia, which lasted for an average of 6.5 minutes (range of 2–12 minutes) and resolved spontaneously or after a vigorous cough. Eight patients (53.3%) had sinus arrest with escaped nodal rhythm and mostly responded to medical treatment (1–2 mg of intravenous adrenaline or atropine). In three symptomatic patients, the escaped rhythm was consistent and followed by early hypoperfusion symptoms and signs and required temporary ventricular pacing. In two cases, the sinus node recovered during a 24-hour follow-up (Table 3). Persistent SNA occlusion followed by progressive hemodynamically instability was reported in one patient, which required a dual chamber permanent pacemaker (Figure 2).

We interrogate the pacemaker twice during a six-month follow-up (after 1 and 6 months from implantation). In the second interrogation, we observe the appearance of the sinus node.

During a median follow-up of 44 ± 5 months, none of the 14 patients who had sinus node occlusion developed sinus node dysfunction. Two patients underwent PCI during the follow-up period in LAD and Cx territories. Angiography of the PCIs revealed patent SNA.

## 4. Discussion

Our study indicates that sinus node artery occlusion during angioplasty involving the RCA's proximal segment and the circumflex artery is rare and occurs in about 5.3% of all PCIs. This incidence is significantly lower than previously reported [5–7]. We believe that the methodology nature of the study could partially explain the low incidence. Previous studies identified sinus node dysfunction in a retrospective fashion. In our study, we reviewed prospectively all PCIs involving the proximal segment to find SNO cases.

The occlusion of the side branch is not necessitating sinus node dysfunction. Still, it has a high potential for developing SND and, in our study, 80% of all SNO cases resulted in some type of SND, which was higher than previously reported. We assume that the significant difference was related to the definition of sinus node dysfunction. In our study, we included all sinus node arrhythmias and, in particular, sinus bradycardia. Since most PCIs in our study involved the inferior wall's infarction, we assume that some cases were related to the phenomenon known as Bezold–Jarisch reflex [16, 17].

All cases of sinus bradycardia following SNO were transient with short duration and resolved uneventfully during the PCI. The appearance of junctional or escaped nodal rhythms following SNO may alert to a more severe and permanent arrhythmia, which will eventually require ventricular pacing.

Out of all SNO events, one patient developed SND, which resulted in hemodynamic instability and required permanent pacemaker implantation. PM interrogation reveals that the sinus node function has the potential to recover in several months.

All SNO has assessed angiography without intracoronary imaging, such as intravascular ultrasound (IVUS) or optical coherence tomography (OCT). We did not use any thrombotic aspiration technique during the PCIs. Therefore, we cannot firmly conclude a relationship between the thrombotic plaque or PCI technique and the appearance of SNO. Moreover, we did not find a statistically significant correlation between stent's length, stent diameter, the formation of thrombus containing lesions, the need for pre- and postballoon dilatation, or TIMI flow due to the appearance of SND (Table 4 and Table 5).

To clearly understand the arrhythmogenic consequences of SNO, we excluded patients with baseline sinus bradycardia; therefore, the impact of nodal sinus dysfunction following SNO in this group remains unclear.

### 4.1. Limitation of the Study

The major limitation of the study is the methodology of the study. Our study is a cohort and retrospective, so we relied only on the quality and views already done. However, our interventional team is small, and there is no significant difference in the catheterization technique. Three senior cardiologists reviewed the cases separately in order not to influence the results. In case of disagreement, we consult all interventionalists.

Given the rarity of the outcome, which was significantly lower than expected, the small study group limits our ability to draw meaningful conclusions regarding risk factors but instead presents a snapshot of the phenomenon.

We collected data in a retrospective manner using Clalit Health Services' computer records, which may underestimate long-term SND's true incidence. Clalit Health Fund is the largest of Israel's four health funds, with more than half of Israel's citizens' members. The HMO's computer system is continuously updating medical information from all medical services, including other health service organizations, and therefore, the risk of missing vital information is significantly low.

## 5. Conclusion

Following angioplasty of the RCA and circumflex arteries' proximal segments, sinus node artery occlusion is rare yet poses a significant risk for developing sinus node dysfunction. In most cases, the SND is uneventful and either resolved spontaneously or with medical treatment, usually within minutes. In only sporadic cases, persistent SND appears and requires the implantation of ventricular pacing. Long-term follow-up reveals that sinus node function in most persistent SND cases tends to recover, mostly within 24 hours from PCI.

Most of the cases of SNO occur during urgent PCI following acute coronary syndrome. We could not conclude or indicate risk factors related to patients' characteristics or PCI techniques and SNO or SND appearance.

## Figures and Tables

**Table 1:** Inclusion and exclusion criteria.

Inclusion criteria	Exclusion criteria
Coronary catheterization during the study period, including elective, urgent, and STEMI PCIs	Evidence of sinus node dysfunction before PCI as follows: 1. Inappropriate sinus bradycardia 2. Bradycardia-tachycardia syndrome 3. Sinus pause or arrest 4. Sinoatrial (SA) exit block 5. Known atrial tachycardia, atrial flutter, or atrial fibrillation before PCI 6. Pacemaker, ICD, and CRT/P implantation

**Table 2:** Patient characteristics.

Patients characteristics	Number (%)
Age (years)	69.58 ± 8.85
Average ± SD, range	43–82
Male	13 (86)
Obesity	6 (40)
Smoker	9 (60)
Hyperlipidemia	15 (100)
Hypertension	11 (73)
Diabetes mellitus	8 (53)
*PCI*	
Elective PCI	2 (13)
Urgent	4 (27)
Primary	9 (60)
*Presentation*	
STEMI	9 (60)
Non-STEMI	4 (27)
*Artery involved*	
Right coronary artery	15 (100)
Circumflex artery	0 (0)

STEMI, ST-elevation myocardial infarction; NSTEMI, non-ST elevation myocardial infarction.

**Table 3:** Detailed information regarding sinus node dysfunction.

	Age	Presentation	SND appearance and type	Duration of SND	Major complains	Clinical outcome
1	64	NSTEMI	Nodal rhythm	10 min	Presyncope	Resolved spontaneously
2	72	NSTEMI	Sinus bradycardia	2 minutes	Dizziness, general weakness	Resolved spontaneously^X^
3	64	STEMI	Nodal rhythm	24 hours	Continuous dizziness, sweating	Temporary PM implantation
4	61	STEMI	Nodal rhythm	1–3 months	General weakness, reduced functional capacity, effort dyspnea	Permanent PM implantation
5	69	STEMI	Nodal rhythm	3 hours	Headache, presyncope	Temporary PM implantation
6	70	STEMI	None			
7	64	STEMI	None			
8	65	STEMI	Nodal rhythm	3 hours	Shortness of breath, sweating	Resolved with medication^Y^
9	78	Elective PCI	Sinus bradycardia	4 minutes	Light-headedness	Resolved spontaneously
10	82	Elective PCI	Nodal rhythm	45 minutes	Presyncope, headache	Resolved with medication^Y^
11	75	STEMI	Nodal rhythm	6 hours	Headache, dizziness	Resolved with medication^Y^
12	43	NSTEMI	None			
13	72	STEMI	Nodal rhythm	1.5 hours	Headache, sweating, weakness	Resolved with medication^Z^
14	52	STEMI	Sinus bradycardia	12 minutes	General weakness	Resolved spontaneously
15	81	NSTEMI	Sinus bradycardia	8 minutes	Dizziness	Resolved spontaneously

^X^Resolved after vigorous cough; ^Y^resolved after the use of 1 mg adrenaline; ^Z^resolved after the use of 1 mg atropine. SND, sinus nodal dysfunction; PM, pacemaker; STEMI, ST-elevation myocardial infarction; NSTEMI, non-ST elevation myocardial infarction.

**Table 4:** Angiographic data of the study population.

	Age	Presentation	SND	Thrombus containing lesion	Balloon predilatation	Balloon postdilatation	Stent^¥^ length	Stent diameter	TIMI flow prestenting	TIMI flow poststenting
1	64	NSTEMI	Nodal rhythm	No	No	No	12	2.0	1	3
2	72	NSTEMI	Sinus bradycardia	No	No	No	16	2.0	1	3
3	64	STEMI	Nodal rhythm	Yes	Yes	Yes	28	2.5	0	3
4	61	STEMI	Nodal rhythm	Yes	Yes	Yes	22	3.0	0	3
5	69	STEMI	Nodal rhythm	No	Yes	Yes	26	2.5	0	3
6	70	STEMI	None	No	No	No	16	2.5	0	3
7	64	STEMI	None	No	No	No	26	3.0	0	3
8	65	STEMI	Nodal rhythm	Yes	Yes	No	14	3.5	0	3
9	78	Elective PCI	Sinus bradycardia	No	No	Yes	16	2.5	2	3
10	82	Elective PCI	Nodal rhythm	No	No	No	20	2.0	2	3
11	75	STEMI	Nodal rhythm	Yes	Yes	Yes	12	2.5	0	3
12	43	NSTEMI	None	No	No	No	14	2.5	0	3
13	72	STEMI	Nodal rhythm	No	No	No	18	3.0	0	3
14	52	STEMI	Sinus bradycardia	No	Yes	Yes	20	3.0	0	3
15	81	NSTEMI	Sinus bradycardia	No	No	No	22	2.5	1	2

^¥^Third-generation drug-eluting stents were used in all procedures.

**Table 5:** Multivariant analysis for SND outcome.

	No SND, *N* = 3 (%)	SND, *N* = 12 (%)	Total, *N* = 15 (%)	*P* value
Age	59 ± 14.17	69.58 ± 8.85	67.47 ± 10.46	0.120
STEMI presentation	2 (66.7)	7 (58.3)	9 (60)	0.744
Thrombus containing lesion	0 (0)	4 (33.3)	4 (26.7)	0.243
Balloon predilatation	0 (0)	6 (50)	6 (40)	0.114
Balloon postdilatation	0 (0)	6 (50)	6 (40)	0.114
Stent length	18.67 ± 6.42	18.83 ± 5.14	18.80 ± 5.171	0.711
Stent diameter	2.67 ± 0.28	2.58 ± 0.46	2.6 ± 0.43	0.709
TIMI flow O prestenting	3 (100)	7 (58.3)	10 (66.7)	0.392
TIMI flow III poststenting	3 (100)	11 (91.7)	14 (93.3)	0.605

## Data Availability

The full data used to support the findings of this study are available from the corresponding author upon request.
